# Cardiac magnetic resonance imaging characterization of acute rejection in a porcine heterotopic heart transplantation model

**DOI:** 10.1371/journal.pone.0304588

**Published:** 2024-06-03

**Authors:** Michelle Mendiola Pla, Carmelo A. Milano, Carolyn Glass, Dawn E. Bowles, David C. Wendell

**Affiliations:** 1 Division of Cardiothoracic Surgery, Duke University Medical Center, Durham, NC, United States of America; 2 Department of Pathology, Duke University Medical Center, Durham, NC, United States of America; 3 Division of Surgical Sciences, Duke University Medical Center, Durham, NC, United States of America; 4 Division of Cardiology, Duke University Medical Center, Durham, NC, United States of America; 5 Duke Cardiovascular Magnetic Resonance Center, Duke University Medical Center, Durham, NC, United States of America; Scuola Superiore Sant’Anna, ITALY

## Abstract

Preclinical disease models are important for the advancement of therapeutics towards human clinical trials. One of the difficult tasks of developing a well-characterized model is having a reliable modality with which to trend the progression of disease. Acute rejection is one of the most devastating complications that can occur following organ transplantation. Specifically in cardiac transplantation, approximately 12% of patients will experience at least one episode of moderate or severe acute rejection in the first year. Currently, the gold standard for monitoring rejection in the clinical setting is to perform serial endomyocardial biopsies for direct histological assessment. However, this is difficult to reproduce in a porcine model of acute rejection in cardiac transplantation where the heart is heterotopically transplanted in an abdominal position. Cardiac magnetic resonance imaging is arising as an alternative for serial screening for acute rejection in cardiac transplantation. This is an exploratory study to create and define a standardized cardiac magnetic resonance screening protocol for characterizing changes associated with the presence of acute rejection in this preclinical model of disease. Results demonstrate that increases in T1 mapping, T2 mapping, left ventricular mass, and in late gadolinium enhancement are significantly correlated with presence of acute rejection.

## Introduction

Over 4,545 heart transplants were performed in 2023 for patients with advanced heart failure (accessed from https://optn.transplant.hrsa.gov/data/view-data-reports/national-data/). Over the last 30 years, the survival rate associated with transplantation has significantly improved [1]. However, acute rejection (AR) remains a major complication conferring substantial morbidity and mortality after transplantation. AR is among the most common complications within the first year after transplantation with 12.6% of recipients experiencing at least one episode of moderate or severe AR within the first year [2]. Incidence of even one episode of AR is a main determinant for increased morbidity post-transplantation.

Preclinical disease models provide a platform to replicate relevant clinical biology that characterizes human disease pathophysiology which can be used for the development of therapeutics. Model translatability ultimately depends on the fidelity observed between preclinical and clinical outcomes [3]. Hence, a crucial aspect of a reliable clinical model is to define methods and parameters to be able to measure the efficacy of an intervention. The porcine abdominal heterotopic heart transplantation model has been described as an ideal model for assessing the immunopathology of acute rejection. It is not reflective of typical cardiac physiology given the offloaded state of the heart as it does not receive a significant portion of blood volume from the recipient. However, it is advantageous over an orthotopic transplantation model due to better overall survival of the recipient. This is because the surgery for transplantation does not require cardiopulmonary bypass intra-operatively, and because there is no requirement of the allograft to maintain the recipient animal’s circulation [4, 5].

Currently, the gold standard for monitoring rejection in the clinical setting is to perform serial endomyocardial biopsies (EMB) for direct histological assessment. However, this is difficult to reproduce in a porcine model of acute rejection in cardiac transplantation where the heart is heterotopically transplanted into the abdomen of the animal. Cardiac magnetic resonance (CMR) imaging is arising as an alternative in the clinical setting for serial screening of acute rejection in cardiac transplantation. CMR has the advantage of being able to screen the entirety of the allograft myocardium, whereas EMB is only able to screen a small, random fraction of it. Evidence of increasing fibrosis, using late gadolinium enhancement, and edema, using T1 and T2 weighted mapping measurements, have been described to correlate with episodes of AR by several clinical studies, potentially making CMR more sensitive for the detection of AR than EMB [6–10].

There is currently no comprehensive or reliable way to monitor transplanted heterotopic abdominal cardiac grafts for AR. Studies that have used this preclinical model have described use of electrocardiography (ECG), cardiac pressure monitoring, myocardial tissue biopsy, blood marker monitoring, and palpation of the graft by physical exam have been described for monitoring for rejection [11–13]. We describe the use CMR to screen transplanted abdominal hearts in a porcine model of acute rejection following heart transplantation and define the changes observed in cardiac activity and morphology using T1 mapping, T2 mapping, and late gadolinium enhancement measurements. We correlated these findings with results from adjunct EMB, blood marker monitoring, echocardiography, and palpation measurements.

## Materials and methods

### Cardiac transplantation and post-operative care

**Fig 1:**
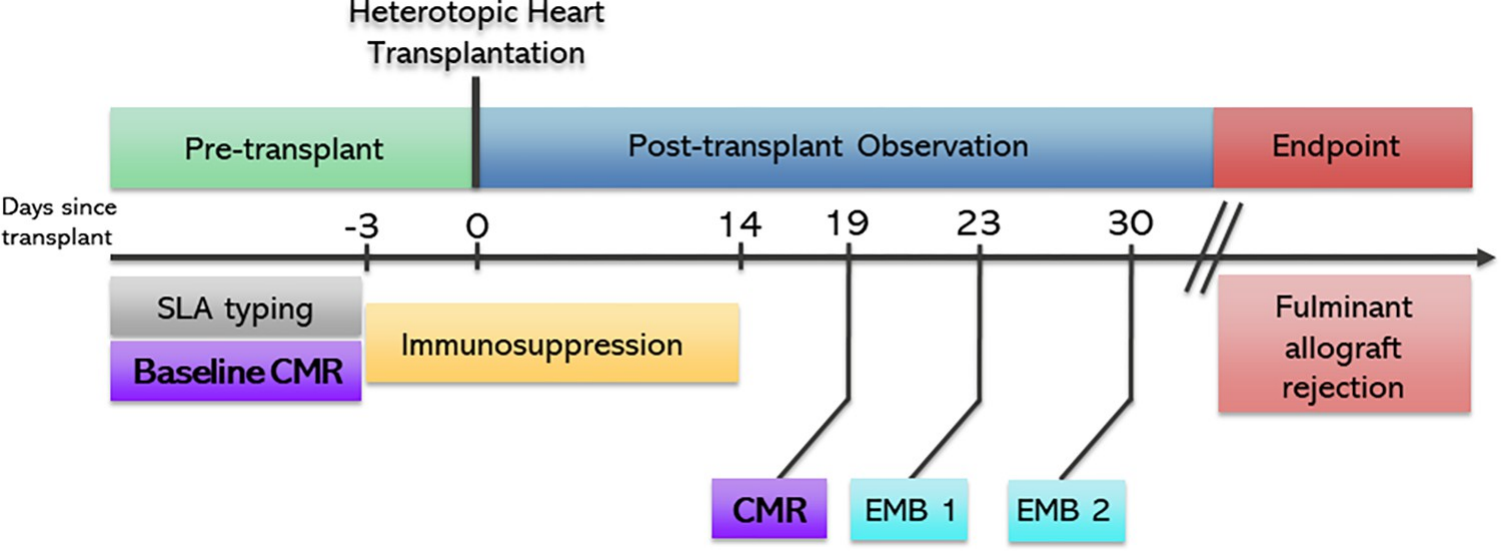
Study timeline of intervention and acute rejection follow-up. Pig pairs underwent a “pre-op” CMR to measure baseline characteristics. Three days prior to abdominal heterotopic cardiac transplantation the pigs began immunosuppression therapy. The pigs then underwent transplantation on post-operative day 0. Immunosuppression therapy ended on post-operative day 14. “Rejection” CMR was performed on post-operative day 19 to assess for early onset of acute rejection in the allograft. Afterwards, transvenous endomyocardial biopsy was performed on post-operative day 23 and 30 in order to validate and trend the progression of acute rejection seen on CMR. After these procedures the cardiac allografts were monitored by physical exam until the allografts reached fulminant acute rejection as determined by all cessation of cardiac activity.

The care and treatment of pigs followed the *Position of the American Heart Association on Research Animal Use* [14]. This study was approved by the Duke University Institutional Animal Care and Use Committee (A122-22-06). Female Yucatan pigs (Sinclair Bio Resources, Auxvasse, MO) aged 7–9 months were used. The weight of the pigs ranged between 13-42kg. All pigs underwent SLA genotyping and blood-typing prior to selection. Pairs that were blood-type compatible and fully SLA Class 1 and Class 2 mismatched were utilized [4, 15–17]. The pig weighing the least in each pair was selected for use as the donor animal. The donor hearts were procured in standard fashion and underwent normothermic ex vivo perfusion using a TransMedics Organ Care System device for 2 hours as described in Mendiola Pla *et al*. [4, 18]. The hearts were subsequently transplanted heterotopically into the recipient pig’s abdomen [4]. The study interventions and follow-up are outlined in Fig 1. For immunosuppression, the recipient pigs began receiving tacrolimus intramuscular injections three days prior to transplantation with a trough goal of 5-15ng/L. Following transplantation, the animals continued receiving tacrolimus injections. They were also started on a methylprednisolone taper and mycophenolate mofetil 400mg twice daily. The immunosuppression was stopped post-operative day (POD) 14 [19].

Animal health and behavior were monitored two to three times a day during the experimental period by trained personnel. The animals were sedated and underwent endotracheal mechanical ventilation for every invasive procedure and for CMR scans. General anesthesia using ketamine (5–33 mg/kg) and midazolam (0.2–0.5 mg/kg) intravenously was used then sedation was maintained using isoflurane gas (1–3%). Buprenorphine (0.005–0.01 mg/kg) was administered intramuscularly post-operatively for analgesia management. Cardiac activity was assessed daily by palpation and graded on a 0–4+ scale, where 0 corresponds to no palpable pulse whereas 4+ corresponds to a vigorous pulse that can be seen through the skin. Troponin blood levels and echocardiography were assessed weekly throughout the survival period.

Animals were survived until fulminant graft rejection was reached as determined by complete cessation of allograft activity on palpation and echocardiography or if allograft survival surpassed greater than 100 days. Once fulminant graft rejection was reached, animals were euthanized within 24 hours. If an allograft survived greater than 100 days, the animals were euthanized within one week of the timepoint. For euthanasia, animals again underwent endotracheal mechanical ventilation and sedation as described above. To procure the hearts, a sternotomy and laparotomy were performed and the hearts were each arrested using Del Nido cardioplegia infused directly into the respective aortic root of each heart. Each heart was subsequently excised from the animal.

### Cardiac magnetic resonance

**Fig 2:**
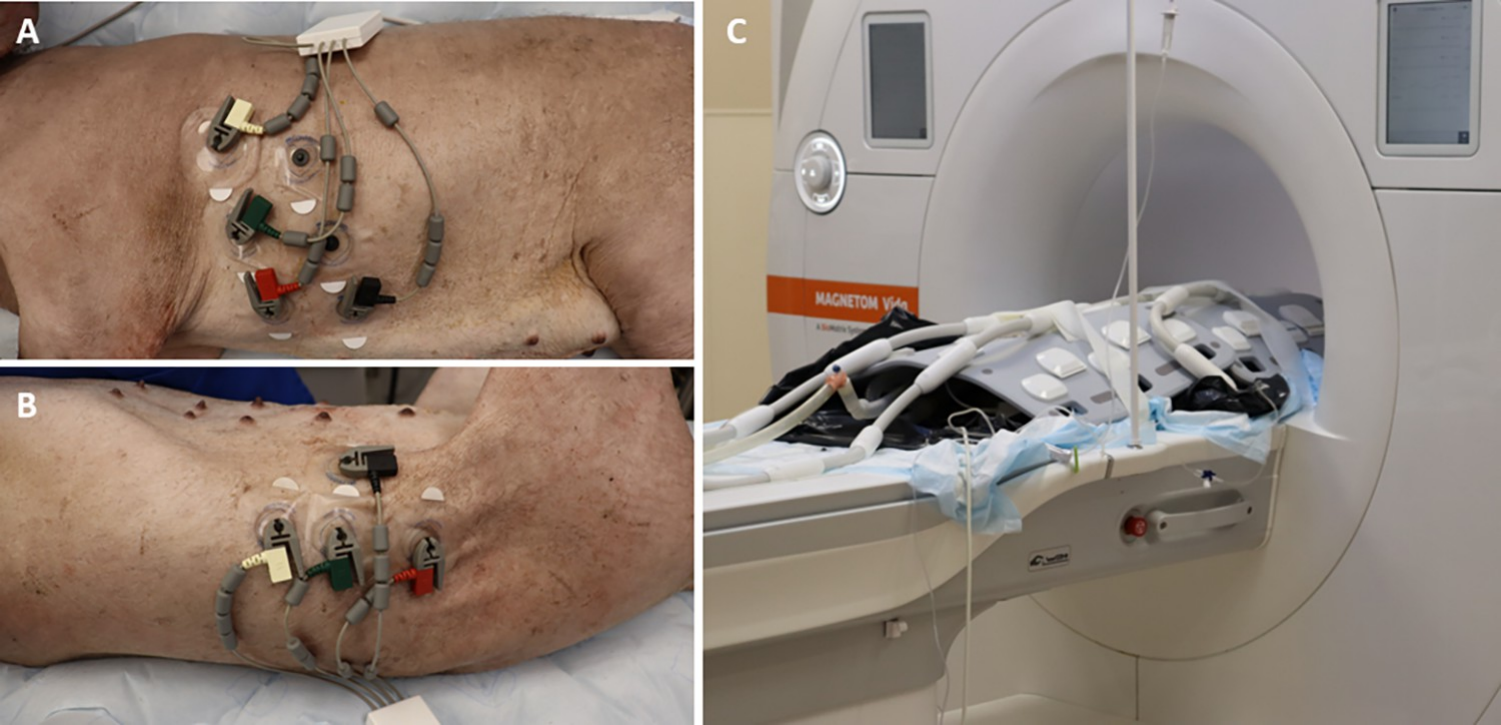
Electrode lead and coil configuration. Electrode lead configurations for obtaining the electrocardiogram measurements from the (A) recipient’s native heart in the thorax and from the (B) heterotopic cardiac allograft in the abdomen. (C) Two coils were used to obtain the CMR images from the native heart and the transplanted heart.

CMR studies were performed using a 3.0T MAGNETOM Vida scanner (Siemens Healthineers, Erlangen, Germany) equipped with a 32-channel chest coil and 72-channel spine array. CMR imaging was performed on both the donor and recipient animal up to 2 weeks prior to cardiac transplantation. Following transplantation another CMR imaging study was performed on POD 19 to characterize changes associated with AR. All pigs underwent sedation with general anesthesia and intubated for mechanical ventilatory support during the duration of the imaging studies. All images were acquired during ventilated breath holds and with cardiac gating applied using electrocardiography (ECG) [20–22]. For the post-transplantation CMR studies, both the native and transplanted heart were able to be gated independently. To achieve this, one set of ECG electrode leads was arranged over the left chest to gate the animal’s native heart and another set was arranged over the right flank (Fig 2). The hearts were imaged sequentially with the ECG gating leads placed first on the chest when imaging the native heart then subsequently moved to the abdominal gating leads when imaging the transplanted heart.

Different imaging techniques were obtained for analysis: Cine-CMR, T1 mapping, T2 mapping, and delayed enhancement CMR (DE-CMR). Cine-CMR was performed using a steady-state free-precession (SSFP) sequence with short-axis images acquired from the level of the mitral valve annulus though the apex (slice thickness 6mm; no gap; temporal resolution: <35ms). T1 mapping images were acquired using 5(3)3 sampling algorithm at slice locations corresponding to cine images. Spatial resolution was 1.0cm x 1.0cm, slice thickness 6mm, and temporal resolution <150ms. T1 mapping raw images were inspected for any mis-registered images, and if necessary, offline T1 mapping analysis was performed. T2 mapping images were also acquired at the same slice locations as cine and T1 mapping images. This sequence consisted of 3 single-shot SSFP images with increasing T2 preparation times (0ms, 25ms, 55ms).

DE-CMR images were obtained 15-minutes after administering gadoterate meglumine (0.15mmol/kg) intravenously. DE-CMR images were obtained using the following parameters: slice thickness: 6mm; in-plane spatial resolution: 1.0mm ×1.0mm; temporal resolution: 180ms; trigger pulse: 2 R-R intervals; breath hold time: 10-15ms. A segmented, inversion-recovery gradient-echo sequence with magnitude and phase-sensitive reconstructions were used for DE-CMR, with inversion time manually selected to null normal myocardium. Finally, velocity encoded images were acquired near the anastomotic sites for the donor heart to verify patent flow to the graft.

### Analysis of cardiac magnetic resonance images

Pre- and post-transplantation measurements obtained from the CMR scans were compared for both the native heart and the donor heart. Cine-CMR images were used to calculate ejection fraction (EF) and left ventricular (LV) mass. T1 and T2 mapping values were quantified according to the Society of Cardiovascular Magnetic Resonance guidelines, avoiding the most subendocardial and subepicardial portions of the heart [23]. DE-CMR images were analyzed quantitatively using a full-width-half-max method [24], and the amount of fibrosis/necrosis was compared with baseline image findings.

#### Tissue biopsy collection

Animals were sedated using general anesthesia and intubated for ventilation. For endomyocardial biopsy, the procedure was performed as described in Mendiola Pla *et al*. on POD 23 and 30 [25]. Briefly, an 8-Fr introducer sheath was inserted into the femoral vein and a 7-Fr bioptome was inserted through the sheath and fluoroscopically guided into the right ventricle of the cardiac allograft to obtain the biopsies. Sharp biopsies were obtained at the time of fulminant allograft rejection at which time the animal was euthanized and the allograft was excised.

### Histology and rejection grading

All biopsies were fixed in 10% neutral buffered formalin prior to paraffin embedding. 5-micron thick sections were prepared and stained with hematoxylin and eosin (H&E) to assess for rejection grade or with Masson’s trichrome stain to assess for fibrosis. Sections were assessed and graded according to ISHLT guidelines by a cardiac pathologist, CG, who was blinded to the CMR results [26].

### Statistics

Statistical analysis was performed using GraphPad Prism 10 Version 10.1.0 (316) (GraphPad Software, Inc. La Jolla, CA). A p-value of <0.05 was considered significant. Normality was assessed using Shapiro-Wilk test. For comparisons between pre-transplantation and post-transplantation measurements with parametric distributions, the changes in mean values of each group were calculated and compared using Student’s t-test. For those with non-parametric distribution, Mann Whitney U test was used. Statistical notations used in the figures: p>0.05, not significant (ns); p<0.05, *; p<0.01, **; p<0.001, ***; p<0.0001, ****.

## Results

Each animal (n = 14) underwent a baseline CMR scan and each transplant recipient animal (n = 6) underwent a rejection assessment CMR scan on POD 19. All CMR scans were able to be fully completed on all animals without any complications. One transplant recipient animal died in the immediate post-operative period from surgical complications related to bleeding and was excluded from analysis. Of the n = 6 transplanted hearts, n = 5 progressed to fulminant rejection; n = 1 did not progress to fulminant AR by POD 106 and was euthanized at that time. The average time to fulminant rejection following transplantation was 51±22 days (mean±SD) (n = 5).

### Baseline CMR

Baseline CMR measurements are listed in Table 1. Native hearts had an average mass of 57.4±9.0g and allograft heart had a mass of 50.3±8.1g (mean±SD). The average ejection fraction for native hearts was 69.3±7.6% and for allograft hearts was 62.5±7.0% (mean±SD). T1 mapping values were 1138±8.9ms for native hearts and 1144±28.7ms for allograft hearts (p = 0.69). T2 mapping values were 36.9±1.3ms for native hearts and 36.8±1.8mg for allograft hearts (p = 0.9). Collectively, the T1 and T2 measurements indicate that there was no evidence of inflammation or edema of the myocardium prior to transplantation. DE-CMR measurements were negative for fibrosis in both native and donor hearts prior to transplantation.

**Table 1:** Baseline CMR characteristics.

	Recipient (n = 6)	Allograft (n = 6)
LV Mass, g (mean±SD)	57.4±9.0	50.3±8.1
Ejection fraction, % (mean±SD)	69.3±7.6	62.5±7.0
T1 mapping, ms	1138±8.9	1144±28.7
T2 mapping, ms	36.9±1.3	36.8±1.8
Delayed enhancement, %	0	0

### Post-transplantation CMR in the setting of rejection

**Fig 3:**
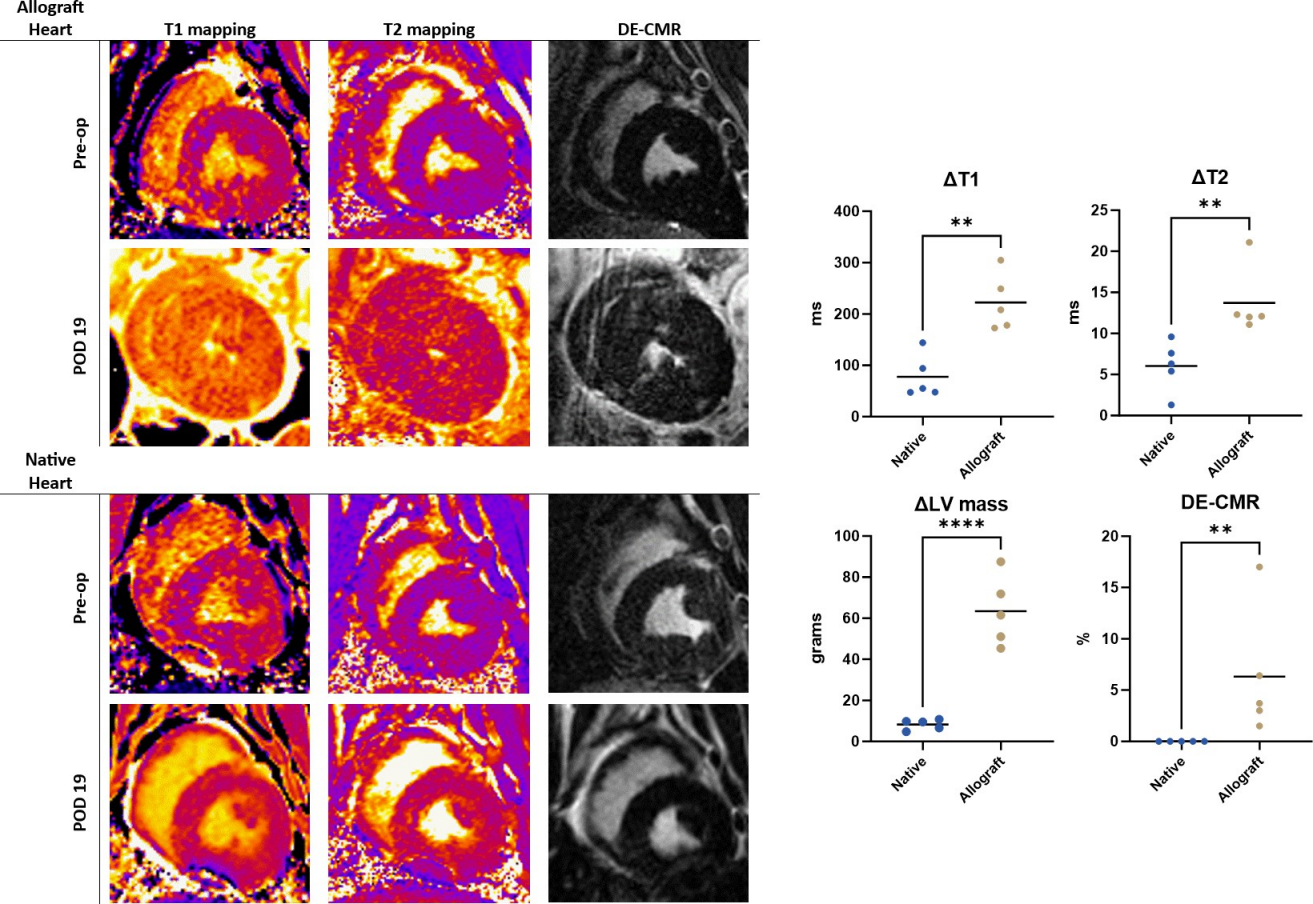
Cardiac magnetic resonance imaging results. Representative CMR T1 mapping, T2 mapping, and DE-CMR images (Animal D) of the native and donor hearts on post-operative day 19. There was marked T1 elevation, T2 elevation, increase in LV mass, and fibrosis as noted by increased DE-CMR percentage. Cumulative results were plotted from ΔT1 measurements, ΔT2 measurements, ΔLV mass, and DE-CMR percentage. (*) <0.05, (**) <0.001, (***) <0.0001.

Post-transplantation CMR results are listed in Table 2 and Fig 3. At 19-days post-transplantation there was significant increase in LV mass in the allograft heart, 63.5±16.8g, compared to the native heart, 8.3±2.5g (p<0.0001). There was also a significant increase in T1 mapping values from baseline in the allograft heart (222.6±55.0ms) relative to the native heart (77.8±41.9ms) (p = 0.002). The increase in T2 mapping values was 6.0±3.1ms for native hearts and 13.7±4.2ms for allograft hearts (p = 0.008). DE-CMR mean percent area of enhancement was 6.5% in the allograft hearts versus 0% in the native hearts (p = 0.008). Velocity measurements across the aortic anastomoses did not show any flow acceleration, demonstrating no areas of inflow stenosis.

**Table 2:** Post-transplantation CMR acute rejection characteristics.

	Native (n = 5)	Allograft (n = 5)	p-value
ΔLV Mass, g (mean±SD)	8.3±2.5	63.5±16.8	<0.0001
ΔT1 mapping, ms	77.8±41.9	222.6±55.0	0.002
ΔT2 mapping, ms	6.0±3.1	13.7±4.2	0.008
Delayed enhancement, %	0	6.3±6.2	0.008

### Post-transplantation CMR in the setting of no rejection

In the animal that did not develop fulminant rejection within the study timeframe, CMR demonstrated an increase of 46.2g in allograft heart LV mass relative to just 10.8g in the native heart. Change in T1 mapping values were in the allograft heart (101.6ms) relative to the native heart (65.5ms). Change in T2 mapping values were slightly increased (9.9ms) in the allograft heart versus (3.1ms) in the native heart. However, both measurements did not reach the levels observed in the animals that did progress to fulminant rejection. Interestingly, DE-CMR demonstrated diffuse and scattered myocardial fibrosis (9.7%) despite other measures of AR being less marked. At the time of donor heart explantation, gross inspection demonstrated small multifocal infarcts scattered throughout the myocardium suggestive of a shower embolic event accounting for this finding on CMR and pathology.

### Supplemental measures of acute rejection

**Fig 4:**
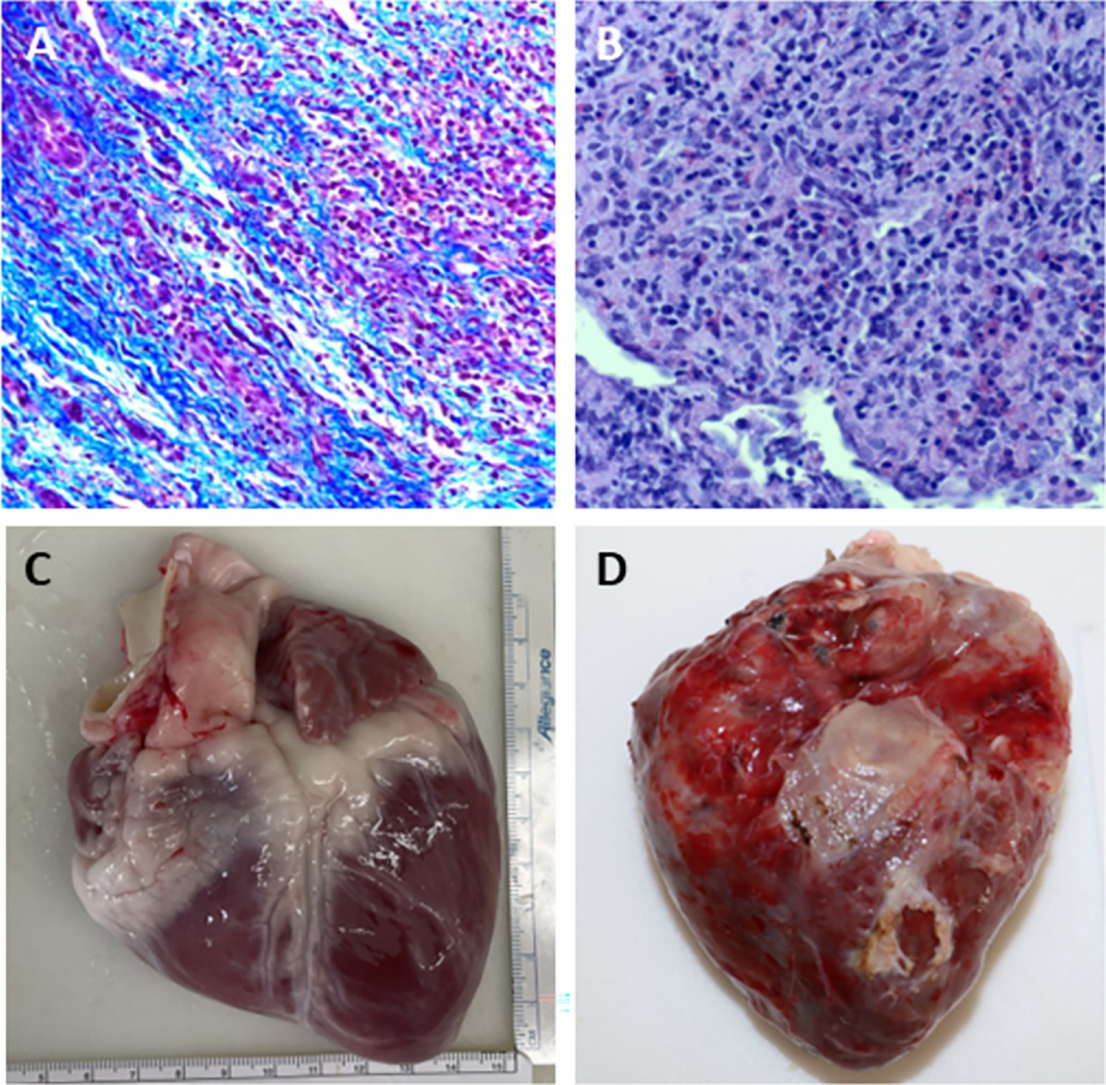
Representative histopathology results of acute rejection. Representative histopathology results of the hearts from one of the transplanted animals (Animal D). Endomyocardial biopsy samples from post-operative day 23 stained for (A) fibrosis (Masson Trichrome) and (B) lymphocytic infiltration (H&E). Both biopsy samples were consistent with acute rejection. At the time of euthanasia on post-operative day 54, the (C) native heart did not show evidence inflammation or scarring, whereas the (D) allograft heart had diffuse inflammation and scarring throughout.

EMB on POD 19 was successfully collected from n = 4 allografts and from n = 3 on POD 30. H&E at POD 19 demonstrated evidence of rejection on n = 2 samples, however no rejection was noted in n = 2 samples (Fig 4A). Of note, one of these samples originated from the allograft that did not reach fulminant rejection prior to the end of study. H&E at POD 30 demonstrated AR on all n = 3 samples obtained. Masson’s trichrome stain on EMBs obtained at POD 19 and POD 30 demonstrated fibrosis in all of the samples (Fig 4B). Average troponin measurements at baseline (221.5±210.7ng/L) were lower than the troponin measurements on POD 19 (7764.4±10398.4ng/L) (mean±SD). At POD 19 the median palpation grade was 3 with an interquartile range of 3 to 3.75. All of the individual supplemental measures are presented in Table 3.

**Table 3:** Correlating measures of allograft injury and rejection.

Animal	Rejection POD 23	Rejection POD 30	Fibrosis POD 23	Fibrosis POD 30	Troponin (ng/L)	Palpation grade	Time to fulminant rejection (days)
A	-	-	-	-	434	3	41
B	-	-	-	-	1458	4	89
C	No rejection	Acute rejection	Fibrosis	-	11185	3	33
D	Acute rejection	Acute rejection	Fibrosis	Fibrosis	1144	3	53
E	Acute rejection	Acute rejection	Fibrosis	Fibrosis	24601	4	39
F	No rejection	-	Fibrosis	-	526	3	n/a

## Discussion

This exploratory study presents the utility of CMR in characterizing AR in a porcine preclinical model of AR in the context of cardiac transplantation, providing comprehensive data for screening for AR [19]. It demonstrates that this modality for examining the allograft is reliable for observing changes in an intra-abdominally transplanted cardiac allograft. This preclinical model of heterotopic heart transplantation is versatile for examining the immunological changes mounted by the recipient and morphological changes that the allograft undergoes at advanced stages of acute rejection [27]. The main limitation of the model across all of these studies is that there is not a highly sensitive method with low associated morbidity that is able to monitor for rejection in the allograft [4, 12, 13, 28]. We used CMR to characterize AR of the allograft on POD 19 after transplantation. Change in LV mass was significantly greater in the donor allograft compared to native hearts. The small increase in mass that is noted in the native hearts is attributed to the expected growth of the pig over the interval period between the baseline CMR and POD 19 CMR. Both T1 and T2 mapping values were each significantly elevated in the donor hearts relative to the native hearts. DE-CMR also demonstrated an increase in fibrotic changes within rejected myocardium that was significant.

The current clinical gold standard for monitoring AR is through EMB. EMB is an invasive procedure that can result in serious complications that compromise the health of the individual. CMR is being used more widely as an alternative diagnostic modality to monitor cardiac allografts post-transplantation. Advances in magnetic resonance imaging technology have allowed for detailed tissue characterization of the heart to be obtained non-invasively with no morbidity associated with the procedure. Many CMR protocols for AR monitoring have been described in the literature and validated against EMB-confirmed incidences of AR. Most clinical studies describe that T2 mapping and ECV values correlate significantly with of AR and while T1 mapping values tended to trend higher they never reached statistical significance in these studies [7, 8, 10]. One study observed that T1 mapping values correlated strongly with episodes of AR and demonstrated correction to baseline values after immunosuppressive therapy [9]. Anthony *et al*. observed that T1 mapping values and T2 mapping values individually correlated with AR, however when combined in a multiparametric fashion the diagnostic sensitivity was higher than either value alone [6]. As described above, our findings agree with the conclusions of these studies that T1 and T2 mapping values become elevated with the presence of AR.

Several techniques have been described previously for monitoring donor graft rejection in this preclinical model. Transabdominal echocardiography is easy to perform where the contractility of the ventricles, LV hypertrophy, and presence of LV thrombus can be assessed. However, consistency of measurements is subject to user variability. Obtaining transabdominal biopsies of the allograft has been used by several groups to be able to monitor the donor graft over multiple timepoints [12, 13]. The transabdominal biopsy technique is limited to one time in these studies since it involves a high risk of injuring the surrounding small bowel or causing significant hemorrhage from the allograft. One group has described the implantation of a telemetry device to measure atrial and ventricular pressures as well as ECG recordings to monitor for rejection [13]. They observed progressive LV hypertrophy and the presence of thrombus on ultrasound in conjunction with increasing troponin and thrombocytopenia, and most definitively an increase in LV end diastolic pressure. However, the technology and environmental setup to be able to use an implantable telemetry device is very expensive and requires a specially outfitted room in order to obtain the recordings. Another group described using daily palpation of the graft with ECG performed twice a week to detect when the heart rate went below 60 beats per minute. Echocardiography was then used to assess systolic function of the graft. Each of these episodes was considered an episode of AR [28]. While this group described this technique to correlate well with AR, this is an indirect method and episodes of bradycardia without continuous monitoring could readily be missed during early onset of AR. Addition of CMR for monitoring the allograft in this model confers advantages, such as the ability to assess the morphology of the heart in a completely non-invasive manner where both the donor and the native heart can be analyzed at the same time.

Serial CMR scans were not able to be performed in each animal, which is a limitation of this exploratory study. As a result, we were unable to characterize the gradual progression of rejection in the allografts towards fulminant rejection within this preclinical model. Future studies utilizing CMR for screening of AR rejection in this preclinical model would be needed to elucidate changes beyond the scope of this proof-of-concept study. However, given the other adjuncts described to monitor rejection, by POD 19 we were able to observe significant changes in the allografts consistent with changes in rejection.

## Conclusion

Application of CMR for monitoring of acute rejection in a porcine model of acute rejection is a safe modality that offers the ability to monitor the entirety of the cardiac allograft and to quantify changes in its functionality. CMR in this setting offers a comprehensive and consistent method for AR monitoring.

## Supporting information

S1 Data (XLSX)

S1 Video Allograft CMR. Pre-op cine clip of the allograft heart followed by the post-op cine clip of the allograft heart.
(3GP)

S2 Video Native CMR. Pre-op cine clip of the recipient’s native heart followed by the post-op cine clip of the native heart.
(3GP)
